# Heterodinuclear zinc and magnesium catalysts for epoxide/CO_2_ ring opening copolymerizations†
†Electronic supplementary information (ESI) available. See DOI: 10.1039/C9SC00385A


**DOI:** 10.1039/c9sc90095k

**Published:** 2019-05-23

**Authors:** Gemma Trott, Jennifer A. Garden, Charlotte K. Williams

**Affiliations:** a Chemistry Research Laboratory , University of Oxford , Mansfield Road , Oxford , OX1 3TA , UK . Email: charlotte.williams@chem.ox.ac.uk; b School of Chemistry , University of Edinburgh , EH9 3FJ , UK

## Abstract

Correction for ‘Heterodinuclear zinc and magnesium catalysts for epoxide/CO_2_ ring opening copolymerizations’ by Gemma Trott *et al.*, *Chem. Sci.*, 2019, DOI: ; 10.1039/c9sc00385a.



## 

In the original manuscript, an error was made in Table 2 for entries 1–3. Incorrect TON and TOF values were reported for compounds **2b**, **2c** and **2e** at a pressure of 1 bar. Also the PCHC selectivity values for **2b** and **2c** at 1 bar were incorrect. The correct TON, TOF and PCHC values for these compounds are shown below:

**Table 2:** Copolymerization reaction conditions^*a*^

Cat.	Catalyst (mol%)	Temp. (°C)	Pressure (bar)	PCHC^*b*^ (%)	TON^*b*^	TOF^*b*^ (h^–1^)	*M*_n_ [*Đ*]^*b*^
**2b**	0.1	120	1	>96	377	435	12 280 [1.04]
							5340 [1.13]
**2c**	0.1	120	1	>93	419	466	14 490 [1.06]
							5930 [1.15]
**2e**	0.1	120	1	>99	430	645	21 760 [1.04]
							9090 [1.15]
**2c**	0.01	120	20	>99	4415	8830	44 400 [1.04]
							21 200 [1.05]
**2c**	0.005	120	20	>99	5435	1359	54 380 [1.04]
							26 550 [1.04]

^*a*^Reactions were carried out in a Parr high pressure vessel with an impeller at 20 bar.

^*b*^See Table 1 and ESI for all data (Fig. S56–S60).

To highlight this update, the sentence in the original manuscript “For the Zn(ii)/Mg(ii) complexes, the best activity value reaches 654 h^–1^ which is at the upper end of values for the low pressure regime.^10,14,15^” on page 7 should now read “For the Zn(ii)/Mg(ii) complexes, the best activity value reaches 645 h^**–1**^ which is at the upper end of values for the low pressure regime.^10,14,15^”

Finally, a corrected version of Fig. 4 is also provided here to highlight the correct TOF value for complex **2e**.

**Fig. 4:**
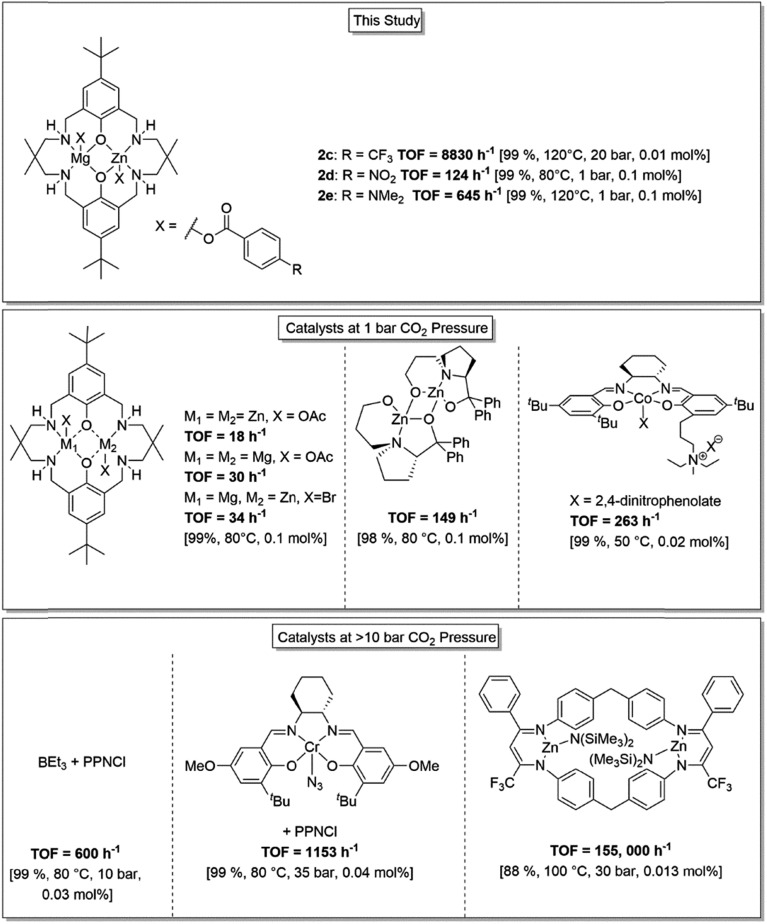
Illustration of the structures, activity and selectivity for some of the highest performing catalysts reported for CO_2_/CHO ROCOP.^22,25,33,34,59,61^

The original ESI was replaced by a correspondingly revised version on 16th May 2019 to reflect these changes.

The Royal Society of Chemistry apologises for these errors and any consequent inconvenience to authors and readers.

